# Health facility assessments of cervical cancer prevention, early diagnosis, and treatment services in Gulu, Uganda

**DOI:** 10.1371/journal.pgph.0000785

**Published:** 2023-02-15

**Authors:** Tana Chongsuwat, Aaliyah O. Ibrahim, Ann E. Evensen, James H. Conway, Margaret Zwick, William Oloya

**Affiliations:** 1 Department of Family Medicine and Community Health, University of Wisconsin-Madison School of Medicine & Public Health, Madison, Wisconsin, United States of America; 2 Gulu Women’s Economic Development & Globalization (GWED-G), Gulu, Uganda; 3 Department of Pediatrics, University of Wisconsin-Madison School of Medicine & Public Health, Madison, Wisconsin, United States of America; PLOS: Public Library of Science, UNITED STATES

## Abstract

**Background:**

Cervical cancer is ranked globally in the top three cancers for women younger than 45 years, with the average age of death at 59 years of age. The highest burden of disease is in low-to-middle income countries (LMICs), responsible for 90% of the 311,000 cervical cancer deaths in 2018. This growing health disparity is due to the lack of quality screening and treatment programs, low human papillomavirus (HPV) vaccination rates, and high human immunodeficiency virus (HIV) co-infection rates. To address these gaps in care, we need to develop a clear understanding of the resources and capabilities of LMICs’ health care facilities to provide prevention, early diagnosis through screening, and treatment for cervical cancer.

**Objectives:**

This project aimed to assess baseline available cervical cancer prevention, early diagnosis, and treatment resources, at facilities designated as Health Center III or above, in Gulu, Uganda.

**Methods:**

We adapted the World Health Organization’s Harmonized Health Facility Assessment for our own HFA and grading scale, deploying it in October 2021 for a cross-sectional analysis of 21 health facilities in Gulu.

**Results:**

Grading of Health Center IIIs (n = 16) concluded that 37% had “excellent” or “good” resources available, and 63% of facilities had “poor” or “fair” resources available. Grading of Health Center IVs and above (n = 5) concluded that 60% of facilities had “excellent” or “good” resources, and 40% had “fair” resources available.

**Discussion:**

The analysis of health facilities in Gulu demonstrated subpar resources available for cervical cancer prevention, early diagnosis, and treatment. Focused efforts are needed to expand health centers’ resources and capability to address rising cervical cancer rates and related health disparities in LMICs. The development process for this project’s HFA can be applied to global cervical cancer programming to determine gaps in resources and indicate areas to target improved health equity.

## Introduction

Globally, cervical cancer is one of the top three cancers in women younger than 45 years, with an average age of death at 59 years of age. The greatest disease burden is in low- and low-to-middle-income countries (LMICs), contributing to 90% of the 341,000 cervical cancer deaths globally in 2020 [1]. About 12% of new cases occur in African women, yet 85% of deaths occur in Sub-Saharan Africa [2].

Uganda has one of the largest documented cervical cancer disease burdens in Africa, with an incidence rate of 47.5 out of 100,000 women and a mortality rate of 40 out of 100,000 women [3]. In Uganda, the Kampala Cancer Registry collected and calculated incidence rates for different cancers from 1991–2006 and found cervical cancer to be the most common malignancy in women with a 3% annual increase in incidence over 16 years [4]. One half (50.1%) of all female cancers in the Acholi Sub-region, which encompasses Northern Uganda, are cervical, with cases most commonly occurring between 30 and 49 years of age an incidence of 57 out of 100,000 women [5].

There are many common challenges for cancer screening and treatment programs in LMICs, including lack of infrastructure and non-surgical treatment modalities, and chronic shortages of health workers and resources. Additionally, weak referral processes and inadequate health information systems make it difficult to track individual patients or monitor program performance [6, 7].

Despite many national health systems prioritizing quality cervical cancer screening programs, these expanded services are significantly underutilized. In Uganda, the health system was purposefully decentralized with the intent of improving access and quality of health services. While this has led to increased utilization of health facilities, it has divided the system into national and district health systems, which have subsequently faced challenges due to pharmaceutical drug shortages, inefficient utilization of resources, and low morale among hospital staff, all of which combine to limit implementation and sustainment of cancer programming [8].

In 2010, the Uganda Ministry of Health developed a Strategic Plan for Cervical Cancer Prevention and Control (2010–2014), with priority areas placed on vaccination against human papilloma virus (HPV), low-cost screening using visual inspection with acetic acid (VIA), and treatment of early dysplasia (cervical intraepithelial neoplasia) using cryotherapy. The Strategic Plan outlines expectations for health facilities, based on their type or level, for what services are expected to be provided. Health facilities are designated as either a health center (levels I to IV, with village health teams designated as level I and serving the smallest target population size) or hospital (general, regional, or national level) by the Ministry of Health determined by the target population size and the overall service capabilities [9].

Improving quality services in Northern Uganda for cervical cancer prevention, early diagnosis, and treatment is essential. The purpose of this project is to develop and conduct a health facility assessment (HFA) to evaluate the cervical cancer resources of Health Centers III (H/C IIIs) and above in Gulu, Uganda. HFAs are often used to gather large-scale data on a country’s health service availability and quality. They are generally administered by trained facilitators and may include materials inventory, interviews with patients and staff, and service observation [10]. Results from this assessment in Gulu will inform potential targeted interventions to fill the gaps in cervical cancer prevention, early diagnosis, and treatment. Moreover, this HFA development process may inform similar processes for global cervical cancer programming to ultimately counteract the increasing disparities in morbidity and mortality experienced by women in LMICs.

## Methods

This cross-sectional study was conducted in Gulu, the second-largest city in Uganda, located in the Northern region. All 23 health facilities located within the city and designated as H/C III and above were eligible, 21 were recruited with 2 refusals citing time constraint or appropriate respondent not available.

### Development of a health facility assessment

In Uganda, health facilities are evaluated through the Results-Based Financing program using the Health Center Quarterly Quality Assessment Tool (HCQQAT). Facilities in Uganda are assessed by level, with higher-level facilities expected to provide more services than lower-level facilities. The score a facility receives on this assessment determines the amount of supplementary funding allotted to it and thus incentivizes the provision of services and good healthcare outcomes. The Results-Based Financing approach has been found to improve service coverage by at least 27%, especially for children and pregnant women [11, 12].

The World Health Organization (WHO), in their evaluation of HFAs, determined that incongruity between assessment designs has limited cross-assessment comparisons of health facilities. To address this, the WHO created the Harmonized Health Facility Assessment (HHFA), a more comprehensive questionnaire designed to be administered either as a whole or as individual modules [13]. We developed a modified HFA using assessment questions from the WHO HHFA cervical cancer questionnaire that was then adapted to the style of the Ugandan HCQQAT to make it contextually relevant for the Ugandan field officers. The tool and description of changes made can be found in S1 File. The modified HFA included questions in three sections: (1) services available, such as the ability to perform certain services related to screening, treatment, and vaccination; (2) facility support for service performance, such as providers trained to perform VIA, HPV diagnostic testing, colposcopy, and excisional or ablative treatments; and (3) available and functioning equipment for the provision of cervical cancer-related services.

### Data collection

This program evaluation did not meet the federal definition of research (pursuant to 45 CFR 26), therefore exempt from IRB review. Local approval was obtained from the District Health Officer through written request as precedence for similar assessments deemed a public health surveillance activity. Gulu Women’s Economic Development & Globalization (GWED-G), a women-led, local grassroots organization dedicated to institutional and technical capacity building in this region, selected four field officers to conduct data collection. These field officers all had previous experience collecting data for facility assessments, such as the Uganda HCQQAT, from the targeted facilities. They received one half-day of classroom training (approximately four hours) which included education on cervical cancer programs, data collection best practices, and how to administer this HFA. As this tool included adaptations (detailed in S1 File) from an existing internationally tested tool (WHO HHFA), additional piloting of the tool was not deemed necessary. Data collection was subsequently performed over six days in October 2021 from all 21 recruited health facilities listed as Health Center III and above within Gulu City. Field officers prioritized gathering data from the facility chair or a supervisor of the maternity ward or outpatient department, depending on availability. Consent to participate was implied by acceptance of the invitation to provide information. This project was conducted during the COVID-19 pandemic therefore all local and national guidelines to prevent spread of COVID-19 were followed, including masking, physical distancing, and self-monitoring for symptoms.

### Statistical analysis

Analysis of collected data was performed through simple statistics using Microsoft Excel. Reported scores for each section and total were cumulated by health facility level. For quality control, if it was reported that the services were available in Section A but equipment was not functioning or available in Section C then this service was deemed unavailable. For example, facilities that reported the ability to provide screening with VIA but did not have acetic acid in stock were not counted as having the resource available. Similarly, field officers were instructed to check functionality of equipment such as the colposcope, loop electrosurgical excisional procedure (LEEP) machine, cryotherapy, and other materials. If the equipment was not functioning, it was determined the facility did not have the service available.

A grading system (Table 1) was developed to determine whether facilities met the expectations outlined by the Uganda Ministry of Health’s Strategic Plan with adequate support to perform such functions. In the Strategic Plan, H/C IIIs and above are expected to provide HPV vaccination and screening by VIA. In addition to vaccination and screening by VIA, Health Center IVs (H/C IVs) and hospitals are expected to provide treatment for early cervical dysplasia [9]. Facilities were determined to have adequate support for screening, diagnostic, and/or treatment services if specific materials were available, including speculums, gloves, and a dedicated gynecological exam table with functioning stirrups. For example, a H/C III that was able to provide both HPV vaccination and screening with VIA or another method but did not have a dedicated gynecological exam table with stirrups received a “good” grade, as more equipment support was needed to comfortably perform a pelvic examination.

**Table 1:** Grading guidelines for health facilities based on Strategic Plan expectations.

Grading	Health Center III	Health Center IV and above
**Excellent**	Able to provide both services (HPV vaccine and screening)	Provides all 3 with adequate support
**Good**	Able to provide both services (HPV vaccine and screening) but more support needed	Provides all 3 services but more support needed
**Fair**	Only able to provide 1 service	Able to provide 2 of 3 services
**Poor**	Unable to provide either HPV vaccines or screening	Not able to provide any services or only 1

## Results

**Fig 1:**
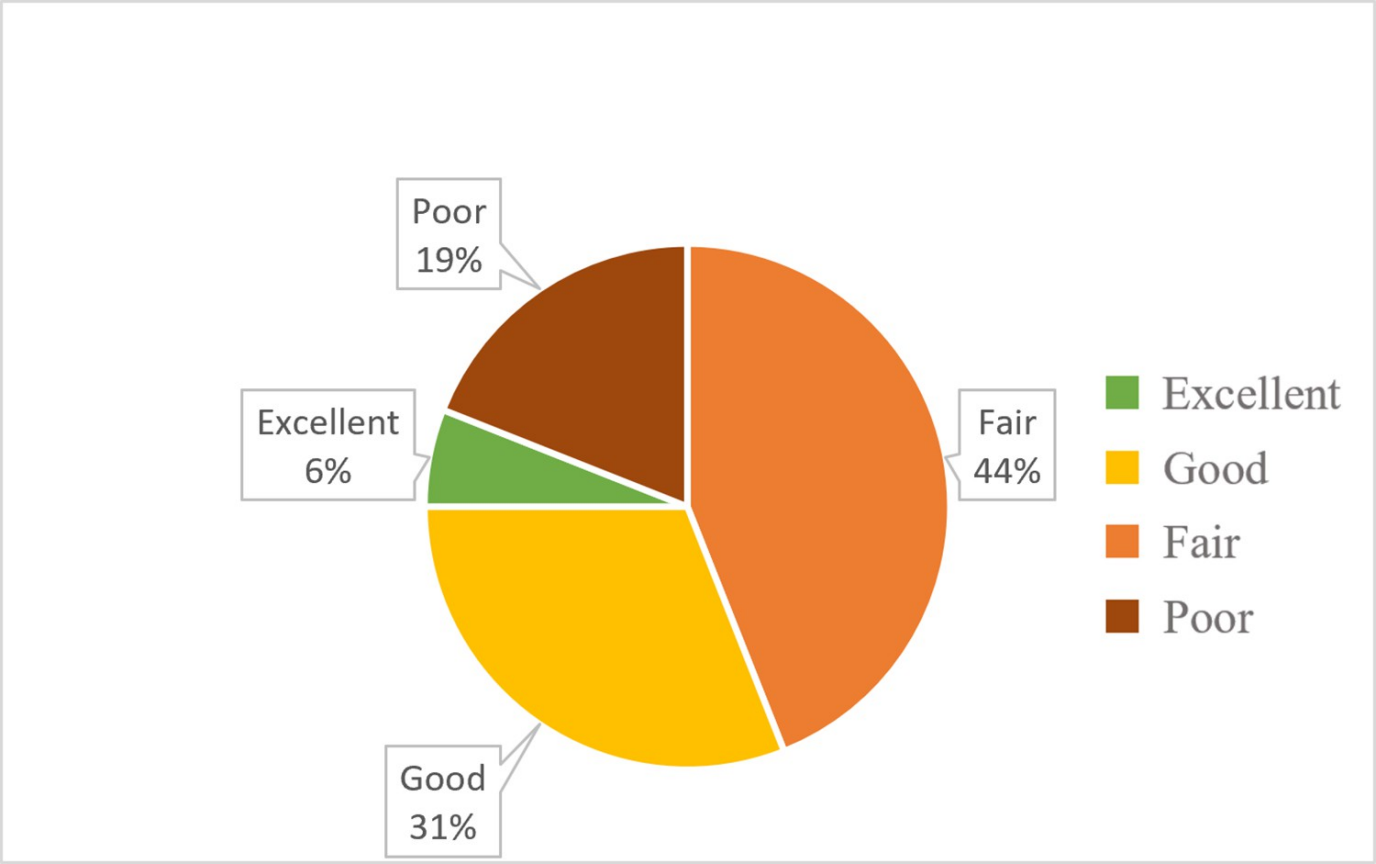
Percentages of Health Center III grading outcomes for assessed cervical cancer services.

**Fig 2:**
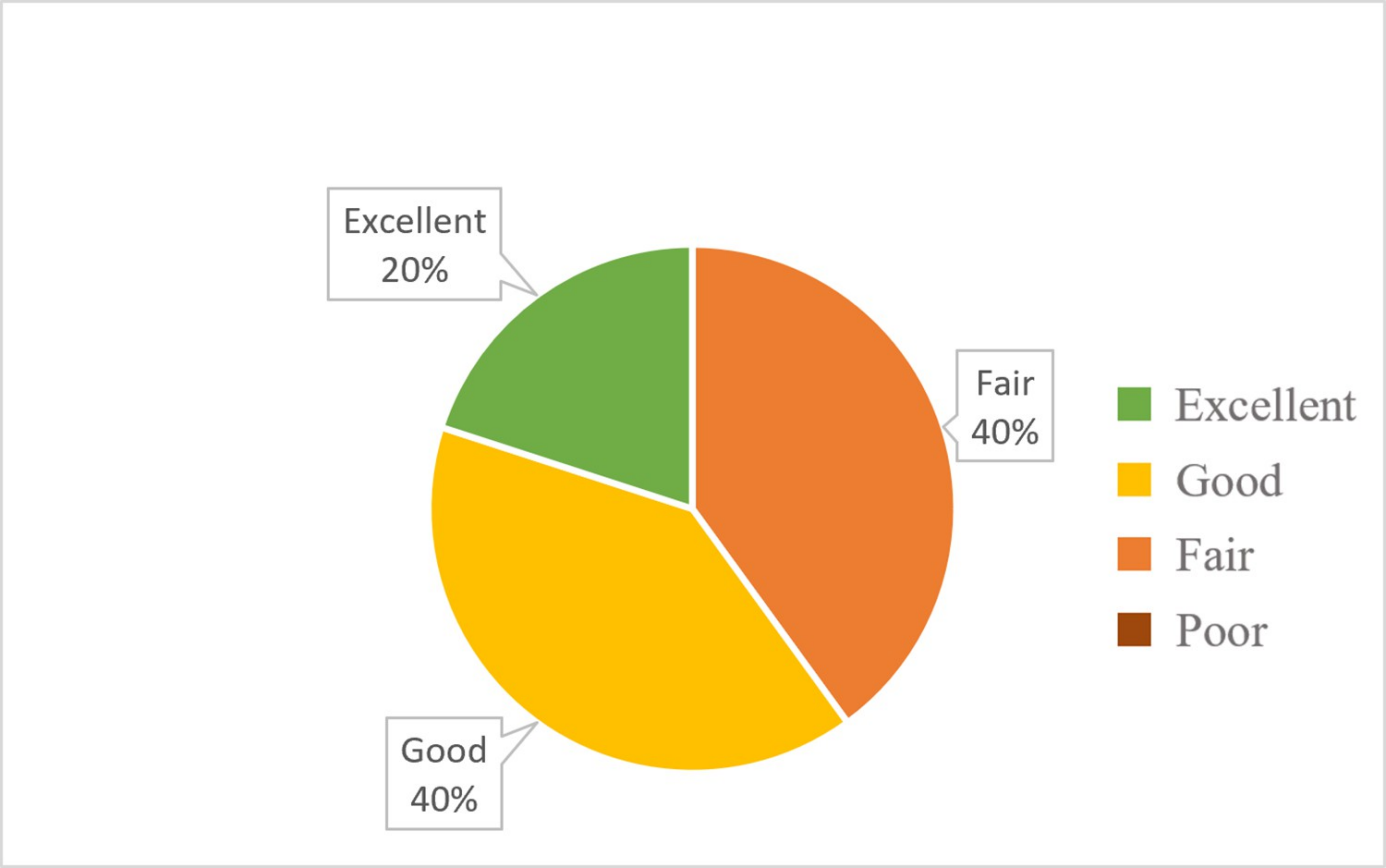
Percentages of Health Center IV and above grading outcomes for assessed cervical cancer services.

Data was collected from 21 facilities that responded to the HFA (16 H/C IIIs, one H/C IV, and four hospitals) and graded based on the guidelines outlined in Table 1. Grading of H/C III facilities (Fig 1) concluded that 37% had “excellent” or “good” resources available, and 63% of facilities had “fair” or “poor” resources available. Grading of facilities H/C IV and above (Fig 2) concluded that 60% had “excellent” or “good” resources, and 40% of facilities had “fair” resources available.

HPV vaccination was available at 62% (n = 13) facilities. VIA was available at 71% (n = 15) of surveyed health facilities. Early treatment for cervical dysplasia was available at six locations, including four of the five facilities where treatment services were expected (H/C IVs and above) and two H/C IIIs. Only one facility was able to provide excisional treatment through LEEP and reported functioning equipment except for a smoke evacuator. Since the procedure could still safely be performed without a smoke evacuator, excisional treatment was included in the resource list for this facility. A summary of resources available by health facility level is included in Table 2.

**Table 2:** List of resources available by facility level.

		Health Center III (n = 16)		Health Center IV and Hospitals (n = 5)		Total (n = 21)	
		n	%	n	%	n	%
**Prevention**							
	Vaccination for HPV	9	56	4	80	13	62
**Screening**							
	Pap Smear (Cytology)	3	19	2	40	5	24
	Visual Inspection with Acetic Acid (VIA)	10	63	5	100	15	71
	HPV PCR testing	0	0	2	40	2	10
**Treatment**							
	Ablation (Cryotherapy)	2	13	4	80	6	29
	Excisional (LEEP ^a^)	0	0	1	20	1	5
**Other Support Needs**							
	Gynecological Bed	8	50	4	80	12	57
	Stirrups	4	25	3	60	7	33
	Biopsy	0	0	3	60	3	14
	Colposcopy	0	0	2	40	2	10

^a^ Loop electrosurgical excision procedure

Overall simple statistical scoring by facility level group is summarized in Table 3 with detailed list available in S1 Data.

**Table 3:** Simple statistical scoring by facility level group.

Facility Group	Health Center III (n = 16)			Health Center IV and above (n = 5)			Maximum Possible Score
	Mean (SD)	Min	Max	Mean (SD)	Min	Max	
Section A: Services Available	2 (1.64)	0	5	6 (1.85)	6	8	9
Section B: Support for Quality Services	2 (1.49)	0	4	4 (1.36)	4	5	5
Section C: Materials	7 (3.98)	0	18	14 (6.92)	14	25	31

## Discussion

To support successful cervical cancer prevention efforts, this project was developed to evaluate health facilities’ current available resources in Gulu, Uganda and provide a systems map for health care workers, public health and policy initiatives, and community members. The data catalogs the resources available in a target geographic region and highlights gaps in resources at facilities that are expected to provide services related to cervical cancer (H/C IIIs and above). Overall, most facilities have available resources to provide comprehensive services, but more support is needed to have dedicated equipment available, trained healthcare workers, and the capacity to provide such services.

Limitations of this project include data collection bias due to ambiguous questions. For example, one item included “Read HPV test,” which was commonly misinterpreted by respondents because it was unclear if this meant ability to run HPV PCR testing on laboratory diagnostic equipment, interpret HPV results, or detect HPV dysplastic changes on the cervix (through VIA, DC, or colposcopy). Based on the Uganda Ministry of Health’s Strategic Plan, expectations of facilities’ ability to provide certain services differed by facility type, although this program evaluation survey used the same HFA for all facilities. Due to these differing expectations, grading proved to be more nuanced than the calculated score. For example, a hospital with an assessment score of 26 points may receive a grade of “poor” overall due to mismatch between available and expected treatment services, while a health center with an “excellent” grade may receive an assessment score of 19 points for providing all basic resources expected of their facility.

Vaccination and early screening through VIA have significantly reduced morbidity and mortality, providing up to 97% relative reduction in cervical cancer rates for girls aged 12–13 years old compared to unvaccinated individuals [14]. Additionally, screening at the age of 35 years using a one- or two-visit screening strategy involving VIA has been found to reduce the risk of cervical cancer by approximately 25% to 36% [15]. While this project and HFA evaluated the availability to HPV vaccines, per recommendations in the Strategic Plan through health facilities, the Uganda national vaccination program focused primarily on girls age 10 to 14 in schools and community outreach programs. Assessing the availability of vaccination in health facilities through this HFA may therefore not accurately capture vaccine accessibility in Uganda.

Cervical cancer screening methods include VIA, cytology, and HPV polymerase chain reaction (PCR) testing. Compared to HPV PCR testing, VIA can be less sensitive but more specific for detecting cervical intraepithelial neoplasia 2+. VIA sensitivity and specificity is approximately 80% (95% CI 0.79–0.82) and 92% (95% CI 0.91–0.92), respectively, compared to HPV PCR Hybrid Capture II (Qiagen, Germany) sensitivity and specificity of 84.0% (95% CI 0.742–0.906) and 88.3% (95% CI 0.818–0.927), respectively [16, 17]. Cytology requires pathologists and/or pathology technicians to examine samples, costly services that are often physically remote from the health center performing the screening. HPV PCR testing is expensive and not widely available. Both cytology and HPV PCR testing require follow-up at a second visit for treatment. Results in this project demonstrate a higher availability of VIA as a screening method in facilities evaluated. Focus for cervical cancer programs in similar settings should support VIA as a low cost and accurate screening method. Training for VIA can be completed in two weeks for nurses and non-physician health care providers, requires very little resources, and promotes screen-and-treat approaches in LMICs.

This HFA evaluated available resources but does not consider if health care workers routinely provide services to patients presenting to health centers requesting or requiring them. Although this project was performed during the COVID-19 pandemic, we did not determine if any infectious control measures, re-allocation of resources, or temporary halt in certain services impacted cervical cancer screening or treatment. A survey of health care workers done prior to the COVID-19 pandemic at H/C IIIs and H/C IVs in Northern Uganda found that only 18% reported being able to conduct screening, and 57% reported relying on outreach from outside organizations for screening, raising concern for a possible disconnect between resource availability and utilization when indicated [18].

In LMICs, different types of health facility assessments are developed and used based on literature reviews, international guidelines, and country specific interests resulting in heterogeneity of indicators and reporting. Overall, similar studies evaluating cervical cancer services in the region have found significant limitations in resource availability and service readiness [19–22]. One study in Malawi published a short report of their 2018–2019 findings of 1106 facilities assessed using the WHO HHFA and determined 69% of hospitals offered cervical cancer services, with greater availability through government hospitals compared to private for-profit hospitals [23]. Efforts to build capacity for cervical cancer control in LMICs are challenged by financing, health care worker shortages, institutional bias, and potential limitations due to the ongoing COVID-19 pandemic. Current roadmaps and evidence-based interventions call for expanded capacity-building efforts, particularly HPV vaccination and same-day screen-and-treat approaches [15, 24].

Using the findings from this project, health facilities, public health professionals, and other policy makers can address the gaps in cervical cancer prevention and control through support of facilities lacking key services or requiring more operational support. The HFA used in this project may not adequately address capabilities of cervical cancer programs therefore further research and development work will be needed to ensure successful implementation and sustainability of programs for long term reduction in cervical cancer morbidity and mortality.

## Conclusion

Our analysis of health facilities in Gulu, Uganda was performed through a modified health facility assessment incorporating assessment questions from the WHO Harmonized Health Facility Assessment tool and utilizing local expertise to administer the survey. Results from 21 of the 23 facilities demonstrated subpar resource availability in the facilities expected to provide cervical cancer prevention, early diagnosis, and treatment services in their communities. Focused efforts on expanding health centers’ resources and capabilities to sustainably provide services will be essential in reducing cervical cancer rates and related health disparities in LMICs.

## Supporting information

S1 File Modified health facility assessment. (DOCX)

S1 Data Cumulative data collected from all health facilities. Highlighted Yellow = Service as expected for the health facility level. Highlighted Orange = Materials needed for adequate support to perform screening, diagnostics, and/or treatment services.
(XLSX)

## Abbreviations

Bx: Biopsy
Colpo: Colposcopy
DC: Digital Cervicography
H/C III: Health center III
H/C IV: Health center IV
HPV: Human papilloma virus
LEEP: Loop electrosurgical excision procedure
Pap: Papanicolaou test
Tx: Treatment
VIA: Visual inspection with acetic acid
VILI: Visual inspection with Lugol’s iodine
