# Supplementary material for: Health facility assessments of cervical cancer prevention, early diagnosis, and treatment services in Gulu, Uganda
Source: PLOS Glob Public Health. 2023 Feb 15;3(2):e0000785. doi: 10.1371/journal.pgph.0000785 (PMC10021907; doi:10.1371/journal.pgph.0000785)
Supplement: S1 File — (DOCX) [file pgph.0000785.s001.docx]

**S1 File: Modified Health Facility Assessment**

Below is the modified health facility assessment used in this study.

**HEALTH FACILITY ASSESSMENT**

**CERVICAL CANCER**

Please find the most knowledgeable person about cervical cancer diagnostic services for the completion of this questionnaire.

Health Facility Name: Date: .

Respondent’s Name: ___________________________ Role____________________________

| **A: Services Available** | | **Assessment**  **If yes 1, if no 0** |
| --- | --- | --- |
| Does this facility have the ability to: | Collect PAP smear specimens |  |
|  | Read PAP smear results |  |
|  | Perform visual inspection with acetic acid (VIA) |  |
|  | Read results for HPV test |  |
|  | Perform colposcopy and biopsy |  |
|  | Read biopsy |  |
|  | Perform digital cervicography |  |
|  | Perform treatment of pre-invasive cervical cancer lesions (e.g. cryotherapy, thermal-cold coagulation or loop electrosurgical excision procedure (LEEP)) |  |
|  | Provide HPV Vaccination |  |

**Total: ____ / 9**

| **B: Support for Quality Services** | **Assessment**  **If yes 1, if no 0** |
| --- | --- |
| Does this facility have any guidelines for cervical cancer screening, diagnosis, or treatment at this service site today?  IF YES, ASK TO SEE THE GUIDELINES |  |
| Have you or any provider(s) received training in VIA in the past 2 years? |  |
| Have you or any provider(s) received training in reading HPV tests in the past 2 years? |  |
| Have you or any provider(s) received training in colposcopy in the past 2 years? |  |
| Have you or any provider(s) received training in performing treatment with LEEP or cryotherapy/thermal-cold coagulation in the past 2 years? |  |

**Total: ____ / 5**

| **C: Materials for Screening, Diagnosis, and Treatment**  **Please tell me if the following basic equipment/items are available in this service area today:**  (ASK TO SEE EACH ITEM THAT IS AVAILABLE) | | **Available?**  **If yes 1, if no 0** | **Functioning?**  **If yes 1, if no 0** | **Quantity** |
| --- | --- | --- | --- | --- |
| Acetic acid for visual inspection (VIA) | |  | n/a | n/a |
| Lugol’s iodine for visual inspection (VILI) | |  | n/a | n/a |
| Speculum | |  | n/a | n/a |
| Glass slides | |  | n/a | n/a |
| Latex gloves | |  | n/a | n/a |
| Goose-neck lamp (or alternative light source – headlamp or flashlight) | |  |  | n/a |
| Gynecological examination table | |  |  |  |
| Stirrups | |  |  |  |
| Digital cervicography equipment | |  |  |  |
| Colposcopy equipment: | Colposcope |  |  |  |
|  | Biopsy forceps or punch biopsy |  |  |  |
| Materials for providing loop electrosurgical excision procedure (LEEP): | LEEP machine |  |  |  |
|  | Smoke evacuator |  |  |  |
|  | Electrosurgical loop electrode |  |  |  |
|  | Coated vaginal speculum (blue) |  |  |  |
| Materials for providing cryotherapy/thermal-cold coagulation | Thermocoagulator or Cryotherapy |  |  |  |
|  | If Cryotherapy -> Nitrous Oxide  *(If equipment does not need nitrous oxide to function, enter “N/A”)* |  |  |  |
| HPV test – Specify type or brand of test________________ | |  |  |  |

**Total: ____ / 31**

**GRAND TOTAL: ­­­­­_____ / 45**

**Reviewer Initials: ______**

The following were added or modified from the WHO HFFA published in March 2021 and used to develop this health facility assessment:

- Section A: Services Available:
  - Addition of “provide HPV vaccination”
- Section B: Support for Quality Services
  - Original question provided in WHO HHFA “Have you or any provider(s) received any training in obtaining cervical specimen procedures or reading HPV tests or visual inspection with acetic acid (VIA) in the past 2 years?”
    - Modified the question for different types of procedures such as VIA, reading HPV tests, colposcopy, and treatment as different items.
- Section C: Materials of Screening, Diagnosis, and Treatment
  - Differentiated VIA and VILI as separate items.
  - Specified materials for providing LEEP as separate items such as: LEEP machine, smoke evacuator, electrosurgical loop electrodes, and coated (blue) speculums.
  - Additional question if cryotherapy was selected as an available equipment included availability of nitrous oxide.
  - Specified type or brand of test for HPV testing
